# PKCθ Synergizes with TLR-Dependent TRAF6 Signaling Pathway to Upregulate MUC5AC Mucin via CARMA1

**DOI:** 10.1371/journal.pone.0031049

**Published:** 2012-01-27

**Authors:** Hirofumi Jono, Jae Hyang Lim, Haidong Xu, Jian-Dong Li

**Affiliations:** 1 Department of Microbiology & Immunology, University of Rochester Medical Center, Rochester, New York, United States of America; 2 Center for Inflammation, Immunity & Infection, and Department of Biology, Georgia State Universtity, Atlanta, Georgia, United States of America; Columbia University, United States of America

## Abstract

CARD-containing MAGUK protein 1 (CARMA1) plays a crucial role in regulating adaptive immune responses upon T-cell receptor (TCR) activation in T cells. Its role in regulating host mucosal innate immune response such as upregulation of mucin remains unknown. Here we show that CARMA1 acts as a key signaling mediator for synergistic upregulation of MUC5AC mucin by bacterium nontypeable *Haemophilus influenzae* (NTHi) and phorbol ester PMA in respiratory epithelial cells. NTHi-induced TLR-dependent TRAF6-MKK3-p38 MAPK signaling pathway synergizes with PKCθ-MEK-ERK signaling pathway. CARMA1 plays a crucial role in mediating this synergistic effect via TRAF6, thereby resulting in synergistic upregulation of MUC5AC mucin. Thus our study unveils a novel role for CARMA1 in mediating host mucosal innate immune response.

## Introduction

The epithelial cells are not merely a passive barrier but can detect foreign pathogens and generate a range of mediators that play important roles in activation of innate and adaptive immunity by recognizing microbial pathogens through surface receptors such as Toll-like receptors (TLRs) [1], [2], [3], [4]. TLRs are type I transmembrane receptors with leucine-rich repeats in the extracellular domain and cytoplasmic domain that resemble the mammalian IL-1 receptor (IL-1R) [3]. To date, 11 members of the human TLR family have been cloned [2], [3], [4]. TLRs have been suggested to play important roles in recognizing microbial components and activating complex signaling networks, which in turn leads to the activation of innate immunity and acquired immunity [3]. There is a growing body of evidence showing that TLR signaling is not only regulated by microbial pathogens, but also modulated by other signaling pathways activated by multiple stimuli, such as growth factors and cytokines [5], [6], [7], [8], [9], [10]. In chronic inflammatory and infectious diseases, multiple inducers, including exogenous and endogenous mediators, are present simultaneously. The molecular mechanisms underlying the regulation of TLR-dependent host mucosal defense response by multiple stimuli, however, remain largely unknown.

Polymeric mucins, the major component of mucus secretions, are high-molecular weight and heavily glycosylated proteins synthesized and secreted by the mucosal epithelial cells lining the middle ear, trachea, and digestive and reproductive tracts [11]. Currently, there are at least 21 unique mucin genes that have been identified and shown to be expressed in tissues, such as ear, lung, nose, salivary glands, and gastrointestinal tracts [11], [12], [13], [14], [15], [16], [17], [18], [19], [20], [21], [22], [23], [24], [25], [26], [27], [28]. Of these, MUC5AC mucin is represents one of the major respiratory mucins [5], [29], [30], [31], [32] and has been shown to be inducible by a wide variety of stimuli, including pro-inflammatory cytokines such as IL-1β, IL-9 and TNF-α, neutrophil elastase, epidermal growth factor receptor (EGFR) ligand, and bacterial pathogens [33], [34], [35]. Mucus production and secretion represents an important host innate defense mechanism in airways by protecting mucus epithelium from microbes, particulates, and other deleterious inhaled substances [12], [36]. However, in chronic disease such as otitis media (OM) and chronic obstructive pulmonary disease (COPD), excess mucus production and hypersecretion is becoming an important pathological factor contributing to morbidity and mortality by causing conductive hearing loss and airway obstruction in OM and COPD, respectively [13], [32], [37], [38], [39]. We previously reported that nontypeable *Haemophilus influenzae* (NTHi), an important gram-negative respiratory pathogen, upregulates MUC5AC expression via TLR2-dependent p38 MAPK signaling pathway [6], [40]. Recently, we have also demonstrated that NTHi and human growth factor EGF synergize with each other to up-regulate MUC5AC mucin transcription [41]. However, how MUC5AC expression is synergistically regulated by multiple stimuli has yet to be fully understood.

Protein kinase C (PKC) is a key modulator in cellular responses mediated by the second messenger diacylglycerol (DAG) and phorbol ester tumor promoters. Activation of PKC leads to a variety of cellular responses such as gene expression, proliferation, and inflammatory and immune response [42], [43]. PKC represents a major family of at least 12 serine/threonine kinases that participate in signal transduction events. PKC isoforms have been classified into three groups: conventional PKCs (cPKC) (α, βI, βII and γ); novel PKCs (nPKC) (δ, ε, η, and θ); and atypical PKCs (aPKC) (ζ and ι/λ). PKC isoforms are widely distributed in mammalian tissues, and certain isoforms are localized to specific tissues to regulate various cellular responses [42]. Recent studies have shown that PKCθ plays a critical role in adaptive immune response by regulating CARMA1 activity, also known as a caspase-recruiting domain 11 (CARD11) [44], [45], [46], [47], [48]. CARMA1 contains a CARD and a membrane-associated guanylate kinase-like (MAGUK) domain and plays a crucial role in regulating adaptive immunity [49], [50], [51], [52], [53], [54], [55]. Activation of PKCθ induces phosphorylation of CARMA1, which in turn leads to activation of NF-κB in T cells [45], [47], [51], [56]. Despite recent studies demonstrating the role of CARMA1 in regulating adaptive immune responses in T cells, its role in regulating innate immune response still remains largely unknown. Moreover, PKC isoforms have been shown to play an important role not only in mucin secretion but also in mucin gene expression [35], [57], [58], [59], [60], [61], [62], [63], [64], [65], [66], [67]. The role of CARMA1 in regulating PKC-mediated mucin gene expression is. However, still unknown.

In the present study we report that phorbol 12-myristate 13-acetate (PMA)-induced activation of PKC synergizes with NTHi-induced TLR2-dependent activation of TRAF6-MKK3/6-p38 MAPK signaling pathway to induce synergistic upregulation of MUC5AC mucin in human respiratory epithelial cells. PKCθ appeasrs to play a critical role in mediating synergistic upregulation of MUC5AC. Moreover, CARMA1 acts as a key signaling mediator downstream of PKCθ to synergistically activate TRAF6. Our studies thus unveil a novel role of PKCθ-CARMA1 pathway in regulating bacterial pathogen-induced TLR-dependent induction of host innate immune response.

## Results

### PMA synergizes with NTHi to induce MUC5AC expression in human epithelial cells

**Figure 1:**
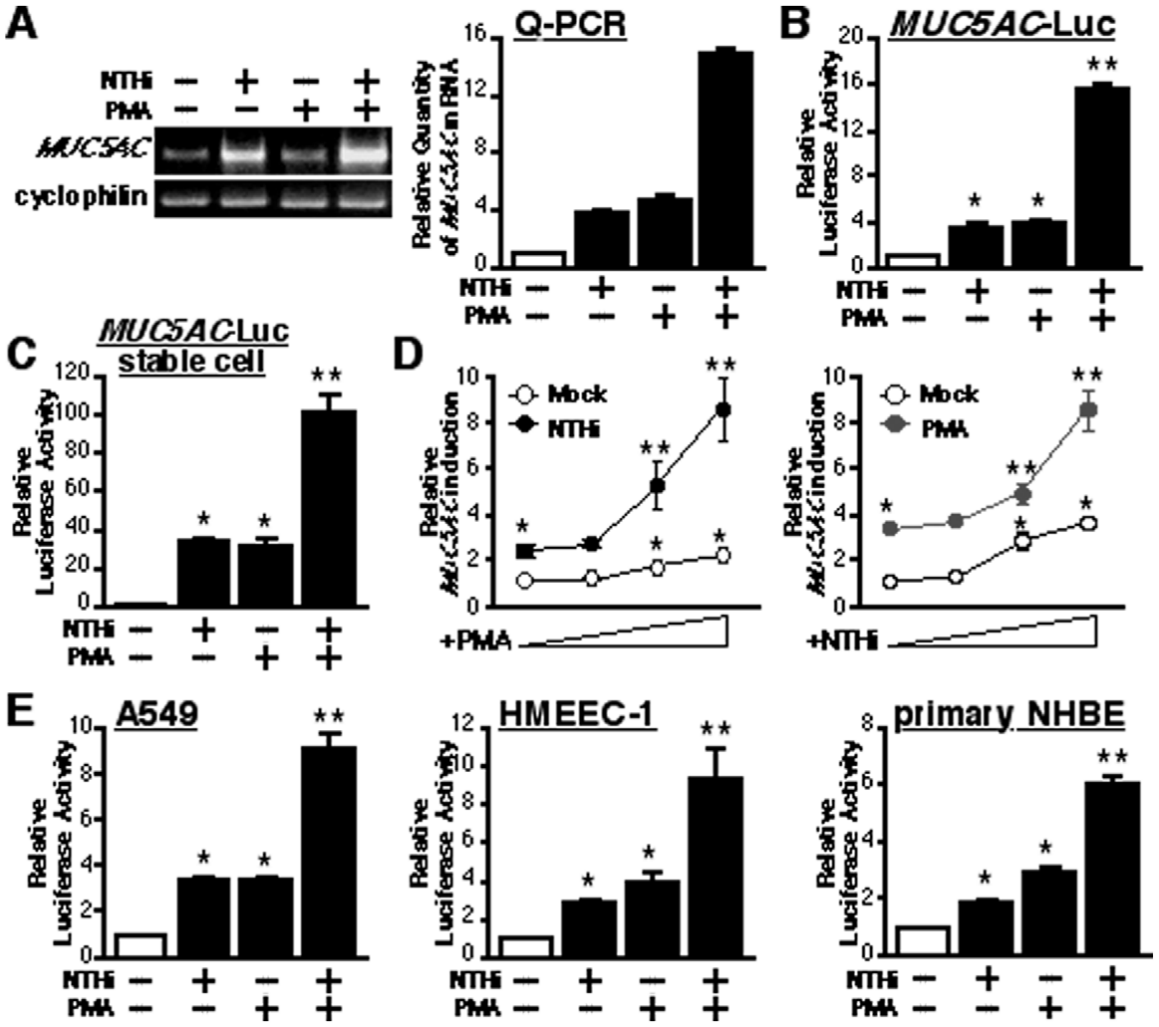
PMA synergizes with NTHi to induce MUC5AC expression in human epithelial cell. (**A**) PMA synergistically enhanced NTHi-induced MUC5AC expression at the mRNA level in human epithelial HM3 cell, as assessed by performing RT-PCR (left panel) and real-time quantitative PCR (Q-PCR) analysis (right panel). Cyclophilin was used as a control for amount of RNA used in each reaction. (**B**) PMA synergized with NTHi to induce MUC5AC expression at the transcriptional level in human epithelial cells, as assessed by MUC5AC-dependent promoter Luciferase assay. (**C**) Synergistic induction of MUC5AC expression by NTHi and PMA was also observed in HM3 cells stably transfected with pMUC5AC 3.7 kb-luc. (**D**) PMA synergizes with NTHi to induce MUC5AC transcription in a dose-dependent manner and vice versa. (**E**) PMA synergistically enhanced NTHi-induced MUC5AC expression at the transcriptional level in human airway A549, middle ear HMEEC-1 and primary bronchial epithelial NHBE cells, as assessed by MUC5AC-dependent promoter assays. Values are the means ± S.D. (n = 3). *p<0.05 vs. control; **p<0.05 vs. NTHi alone. The data shown are representative of three independent experiments. −, absence of; +, presence of; NTHi, nontypeable *Haemophilus influenzae*.

Phorbol esters, such as PMA, have been reported to modulate diverse cellular responses. It has also been reported that PMA not only induces mucin secretion [57], [58], [59], [60], [61], [62], [63], [64], [65], [66], but also induces mucin gene expression [35], [63], [67]. Previously, we showed that NTHi, an important human respiratory pathogen, induces upregulation of MUC5AC mucin, a primary innate defense response in mammalian airways [6], [40], [68]. To determine whether PMA modulates NTHi-induced MUC5AC expression, we first evaluated the effect of PMA on NTHi-induced MUC5AC expression at mRNA level in human epithelial cells, as assessed by performing real-time quantitative PCR (Q-PCR) analysis. As shown in **Fig. 1A**, PMA synergistically enhanced NTHi-induced MUC5AC expression at mRNA level in human epithelial HM3 cells. To investigate whether transcriptional regulation is involved in the synergistic MUC5AC induction, we next transfected HM3 cells with an expression vector containing the human MUC5AC 5′-flanking region fused to a luciferase reporter gene. As shown in **Fig. 1B**, PMA synergized with NTHi to induce MUC5AC expression at the transcriptional level in human epithelial cells. The synergistic enhancement of NTHi-induced MUC5AC transcription by PMA was also observed in HM3 cells stably transfected with pMUC5AC 3.7kb-luc (**Fig. 1C**). Furthermore, PMA synergistically enhanced NTHi-induced luciferase activity driven by the MUC5AC promoter in a dose-dependent manner, and vice versa (**Fig. 1D**), suggesting the involvement of transcriptional regulation. Because we were interested in the potential generality of MUC5AC up-regulation, we assayed a variety of human MUC5AC-expressing epithelial cell lines as well as primary human airway epithelila cells. Consistent with our finding in HM3 cells, PMA synergistically enhanced NTHi-induced MUC5AC expression at the transcriptional level in human airway A549, middle ear HMEEC-1 and primary bronchial epithelial NHBE cells, as assessed by MUC5AC-dependent promoter assays (**Fig. 1E**). Thus, it is evident that PMA synergizes with NTHi to induce MUC5AC expression in multiple human epithelial cells.

### PMA synergistically enhances NTHi-induced MUC5AC expression via MKK3/6-p38 MAPK pathway

**Figure 2:**
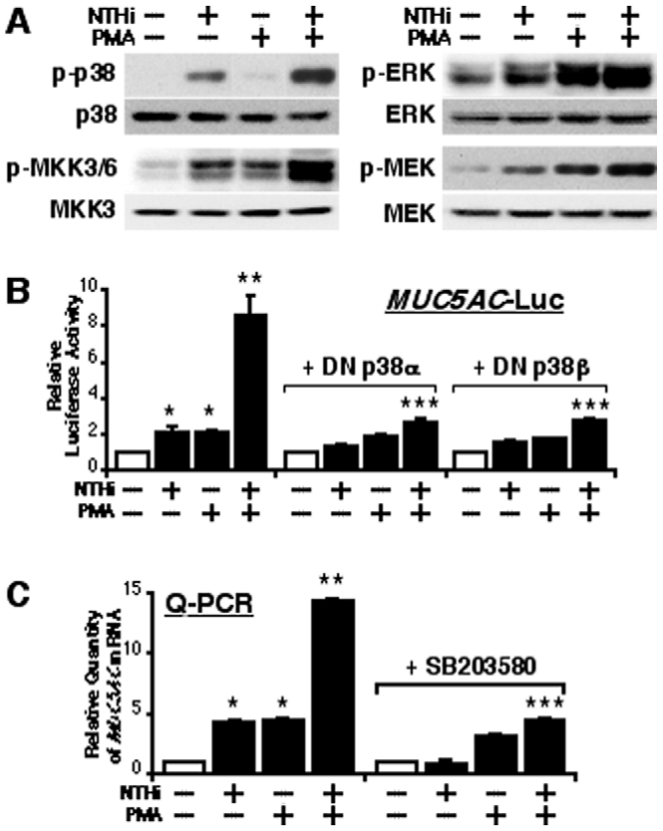
PMA synergistically enhances NTHi-induced MUC5AC expression via MKK3/6-p38 MAPK pathway. (**A**) PMA synergistically enhanced NTHi-induced phosphorylation of p38 MAPK and MKK3/6, but not ERK and MEK1. (**B**) The synergistic induction of MUC5AC transcription by NTHi and PMA was inhibited by overexpressing DN mutant forms of p38α and p38β in human epithelial cells, as assessed by MUC5AC-dependent promoter Luciferase assay. Cells were transfected with 0.8 µg of DN p38α, DN p38β, or control vector, and treated with NTHi with or without PMA. Relative luciferase activity of MUC5AC was measured from the cell lysate. (**C**) SB203580, a specific inhibitor for p38 MAPK signaling, attenuated the synergistic induction of MUC5AC expression by NTHi and PMA at the mRNA level as assessed by Q-PCR. Cells were pre-treated with 10 µM of SB203580 or vehicle control, and treated with NTHi with or without PMA. mRNA expression level of MUC5AC was measured by Q-PCR. Values are the means ± S.D. (n = 3). *p<0.05 vs. control; **p<0.05 vs. NTHi alone; ***p<0.05 vs NTHi with PMA in control vector transfected (**B**) or vehicle treated (**C**) cells. The data shown are representative of three independent experiments. −, absence of; +, presence of; DN, dominant negative; NTHi, nontypeable *Haemophilus influenzae*.

We next sought to investigate the signaling mechanism by which PMA synergistically enhances up-regulation of MUC5AC by NTHi. Based on our recent reports that p38 MAPK, a major MAP kinase superfamily member, has been shown to be involved in NTHi-induced MUC5AC up-regulation [6], [40], [68], it is plausible that activation of p38 MAPK may also play an important role in the synergistic induction of MUC5AC transcription by NTHi and PMA. To determine the involvement of p38 MAPK in PMA-induced synergistic enhancement of MUC5AC expression, we first confirmed the effect of PMA on NTHi-induced phosphorylation of p38 MAPK. As shown in **Fig. 2A**, PMA synergistically enhanced NTHi-induced phosphorylation of p38 MAPK and MKK3/6, its known immediate upstream activators (left panel), but not MEK1-ERK, another major MAPK pathway known to be activated by PMA (right panel). We then determined whether activation of p38 is required for synergistic induction of MUC5AC by assessing the effects of perturbing p38 signaling by co-expressing dominant-negative (DN) mutant forms of p38α and p38β. As shown in **Fig. 2B**, perturbing p38 signaling greatly inhibited the synergistic induction of MUC5AC at the transcriptional level. Consistent with this result, inhibition of p38 signaling using SB203580, a specific inhibitor for p38 MAPK signaling, also attenuated the synergistic induction of MUC5AC expression at the endogenous mRNA level (**Fig. 2C**). Together, these data indicate that MKK3/6-p38 MAPK signaling pathway mediates the synergistic induction of MUC5AC by NTHi and PMA.

### TLR2 is involved in the synergistic enhancement of NTHi-induced MUC5AC expression by PMA

**Figure 3:**
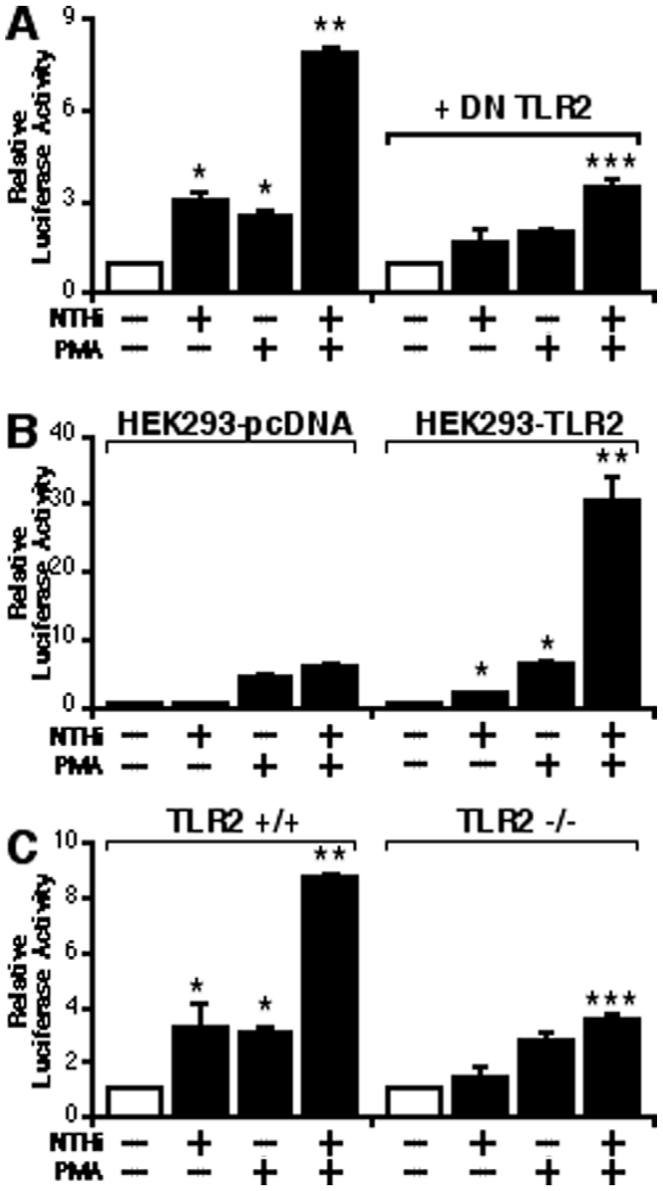
TLR2 is involved in the synergistic enhancement of NTHi-induced MUC5AC expression by PMA. (A) Overexpression of a DN mutant form of TLR2 inhibited the synergistic induction of MUC5AC transcription by NTHi and PMA in human epithelial cells, as assessed by MUC5AC-dependent promoter Luciferase assay. Cells were transfected with 0.8 µg of DN TLR2 or control vector, and treated with NTHi with or without PMA. Relative luciferase activity of MUC5AC was measured from the cell lysate. (**B**) The synergistic enhancement of NTHi-induced MUC5AC transcription by PMA was observed in HEK293-TLR2 cells, but not in HEK293-pcDNA cells. (**C**) PMA did not synergize with NTHi to induce MUC5AC expression in TLR2^−/−^ MEF cells. Values are the means ± S.D. (n = 3). *p<0.05 vs. control; **p<0.05 vs. NTHi alone; ***p<0.05 vs NTHi with PMA in control vector transfected cells (**A**) or TLR2^+/+^ cells (**C**). The data shown are representative of three independent experiments. −, absence of; +, presence of; NTHi, nontypeable *Haemophilus influenzae*; DN, dominant negative; +/+, wild-type; −/−, knock-out.

Having identified that NTHi and PMA synergistically induce MUC5AC up-regulation via MKK3/6-p38 MAPK pathway, still unknown is which cell surface receptor(s) is involved in transmitting signals from the cell surface to the cytoplasm to induce the synergistic upregulation of MUC5AC. Because of the important roles of TLR2 in mediating host response to NTHi [5], [6], [68], we investigated whether TLR2 is involved in the synergistic induction of MUC5AC by NTHi and PMA. As shown in **Fig. 3A**, overexpression of a DN mutant form of TLR2 inhibited the synergistic induction of MUC5AC transcription by NTHi and PMA, suggesting the critical role of TLR2 in mediating synergistic induction of MUC5AC. We next assessed the synergistic enhancing effect of PMA on NTHi-induced MUC5AC expression in HEK293-pcDNA and HEK293-TLR2 cells, which were stably transfected with pcDNA or TLR2, respectively. As expected, the synergistic enhancement of NTHi-induced MUC5AC transcription by PMA was observed in HEK293-TLR2 cells, but not in HEK293-pcDNA cells (**Fig. 3B**). To further confirm the requirement of TLR2 in mediating the synergistic induction of MUC5AC, we next evaluated the effect of PMA on NTHi-induced MUC5AC transcription in WT and TLR2^−/−^ mouse embryonic fibroblasts (MEFs). As shown in **Fig. 3C**, PMA synergized with NTHi to induce MUC5AC expression in WT but not in TLR2^−/−^ MEF cells. Collectively, these data demonstrate that TLR2-dependent signaling is required for the synergistic induction of MUC5AC by NTHi and PMA.

### PMA synergistically enhances up-regulation of MUC5AC induced by activation of TLR signaling

**Figure 4:**
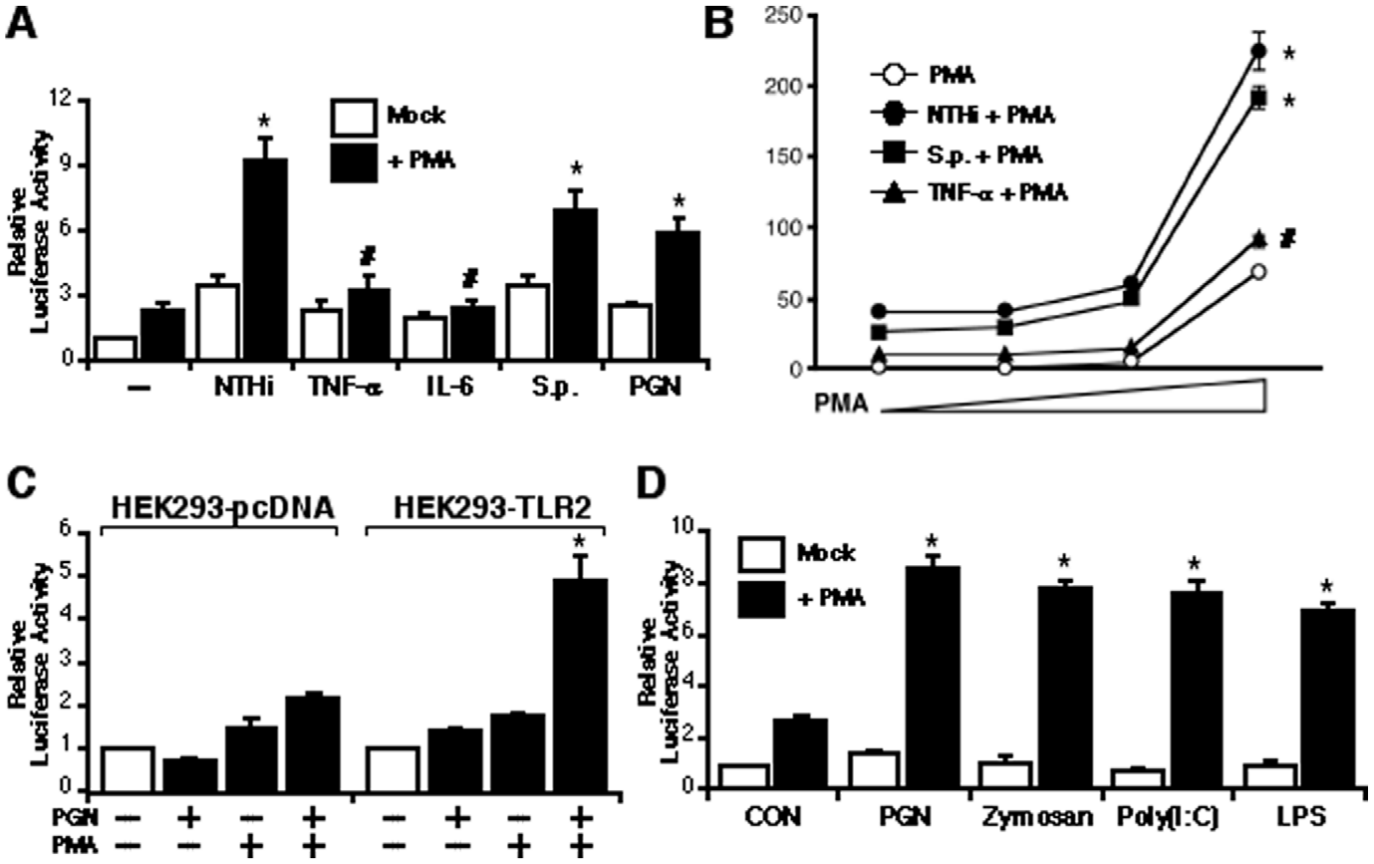
PMA synergistically enhances up-regulation of MUC5AC induced by activation of TLR signaling. (**A**) PMA synergistically enhanced MUC5AC expression induced only by NTHi, *S.p.* and PGN, but not by TNF-α and IL-6, as assessed by MUC5AC-dependent promoter Luciferase assay. Values are the means ± S.D. (n = 3). *p<0.05 vs. NTHi, *S.p.*, or PGN alone; *^#^p>0.05* vs. TNF-α or IL-6 alone. (**B**) PMA synergized with either NTHi or *S.p.*, but not with TNF-α to enhance MUC5AC expression in a dose-dependent manner. Values are the means ± S.D. (n = 3). *p<0.05 vs. PMA alone; *^#^p>0.05* vs. PMA alone. (**C**) PGN-induced MUC5AC transcription was synergistically enhanced by PMA in HEK293-TLR2 cells, but not in HEK293-pcDNA cells. Values are the means ± S.D. (n = 3). *p<0.05 vs. PGN alone. (**D**) Synergistic enhancement of MUC5AC expression by PMA was also observed in the cells treated with TLR ligands, such as PGN, Zymosan, Poly(I:C) and LPS, respectively. Values are the means ± S.D. (n = 3). *p<0.05 vs. PGN, Zymosan, Poly(I:C), or LPS alone. The data shown are representative of three independent experiments. −, absence of; +, presence of; *S.p.*, *Streptococcus pneumoniae*; NTHi, nontypeable *Haemophilus influenzae*.

Because MUC5AC gene expression is up-regulated not only by bacterial pathogens through TLR-dependent pathway, but also by a variety of other stimuli such as pro-inflammatory cytokines [41], [69], [70], [71], we next determined whether PMA can also synergize with the other mucin inducers to up-regulate MUC5AC expression. Interestingly, the synergistic enhancement of MUC5AC expression was observed only in the cell treated with various bacterial stimuli such as NTHi, *Streptococcus pneumoniae* (*S.p.*) and peptidoglycan (PGN), but not in the cell treated with non-TLR stimuli such as cytokines (**Fig. 4A**). In addition, PMA synergistically enhanced MUC5AC expression induced by either NTHi or *S.p.* in a dose-dependent manner, but not by pro-inflammatory cytokine TNF-α, suggesting that the synergistic effect of PMA may be specific only for TLR-dependent MUC5AC up-regulation (**Fig. 4B**). To further determine whether PMA specifically synergizes with TLR-dependent pathway, we next assessed the synergistic effect of PMA on MUC5AC induction by the TLR2 ligand PGN in HEK293-pcDNA and HEK293-TLR2 cells. As shown in **Fig. 4C**, the synergistic enhancement of PGN-induced MUC5AC transcription by PMA was observed in HEK293-TLR2 cells, but not in HEK293-pcDNA cells. Similarly to PGN, the other TLR2 ligand, Zymosan-induced MUC5AC expression was also synergistically enhanced by PMA (**Fig. 4D**). Furthermore, PMA can also synergistically enhance MUC5AC induction by the other TLR ligands, such as TLR3 ligand Poly(I:C) and TLR4 ligand LPS (**Fig. 4D**), indicating that the synergistic effect of PMA may be specific not only for TLR2, but also for the other TLRs-dependent signaling pathway. Thus, these data suggest that PMA synergistically enhances up-regulation of MUC5AC induced by activation of TLR signaling.

### PMA synergistically enhances TLR-dependent MUC5AC induction by NTHi via activation of TRAF6

**Figure 5:**
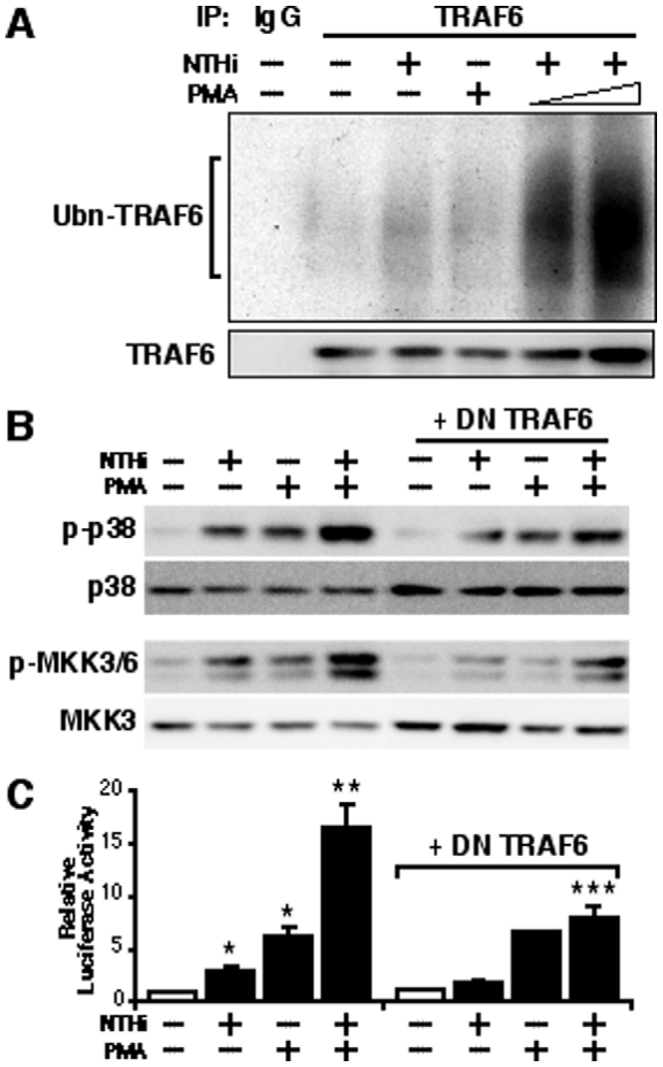
PMA synergistically enhances TLR-dependent MUC5AC induction by NTHi via TRAF6. (**A**) PMA synergistically enhanced NTHi-induced polyubiquitination of TRAF6 in human epithelial cells. Cells were transfected with TRAF6, and were treated with NTHi and PMA as indicated. Whole cell extracts were subjected to co-immunoprecipitation (IP) with either control IgG or anti-TRAF6 antibodies and immunoblotting with anti-ubiquitin antibody. The same blots were re-probed with anti-TRAF6. (**B**) Overexpression of a DN mutant form of TRAF6 blocked the synergistic phosphorylation of p38 MAPK and MKK3/6 induced by NTHi and PMA. Cells were transfected with 0.8 µg of DN-TRAF6 or control vector, and treated with NTHi with or without PMA. Cell lysate was blotted with antibodies as indicated in the figure. (**C**) The synergistic enhancement of NTHi-induced MUC5AC transcription by PMA was inhibited by overexpression of DN-TRAF6 in human epithelial cells, as assessed by MUC5AC-dependent promoter Luciferase assay. Cells were transfected with 0.8 µg of DN-TRAF6 or control vector, and treated with NTHi with or without PMA. Relative luciferase activity of MUC5AC was measured from the cell lysate. Values are the means ± S.D. (n = 3). *p<0.05 vs. control; **p<0.05 vs. NTHi alone; ***p<0.05 vs. NTHi with PMA in control vector transfected cells. The data shown are representative of three independent experiments. −, absence of; +, presence of; NTHi, nontypeable *Haemophilus influenzae*; DN, dominant negative; Ubn, ubiquitin.

One key question that has yet to be addressed is which signaling adaptor molecules transduce signal to mediate the synergistic induction of MUC5AC by NTHi and PMA. In review of all known shared signaling transducers downstream of TLRs, the TRAFs, a family of adaptor proteins, have been shown to be critically involved in the activation of p38 MAPK triggered by TNF receptor and TLR family members [72]. Among all six TRAFs identified previously, TRAF2 has been shown as a signal transducer associated with TNF receptors, whereas TRAF6 has been known to be associated with TLRs [72], [73], [74], [75], [76], [77], [78]. Because our results in **Fig. 4** indicate that the synergistic effect of PMA is specific for only TLR-dependent pathway, we postulated that PMA may mediate its synergistic effect on TLR-dependent signaling via TRAF6. On the basis of evidence that TRAF6 polyubiquitination has been shown to play an important role in regulating TRAF6 activation [73], we first examined whether PMA synergistically enhances NTHi-induced polyubiquitination of TRAF6. Interestingly, NTHi markedly induced polyubiquitination of TRAF6 together with PMA treatment in a dose-dependent manner, suggesting the synergistic effect of PMA on TLR-dependent signaling may occur at the level of TRAF6 (**Fig. 5A**). To further determine whether TRAF6 plays an important role in the synergistic effect of PMA, we next evaluated the effect of perturbing TRAF6 by overexpressing a DN mutant form of TRAF6. As shown in **Fig. 5B**, overexpression of DN-TRAF6 potently inhibited the synergistic phosphorylation of p38 MAPK and MKK3/6 induced by NTHi and PMA. Consistent with these findings, the synergistic enhancement of NTHi-induced MUC5AC transcription by PMA was attenuated by overexpression of DN-TRAF6 in human epithelial cells, as assessed by MUC5AC-dependent promoter Luciferase assay (**Fig. 5C**). Taken together, these data suggest that PMA synergistically enhances TLR-dependent MUC5AC induction by NTHi via TRAF6.

### TLR-TRAF6-dependent synergistic induction of MUC5AC by NTHi and PMA is mediated by PKCθ

**Figure 6:**
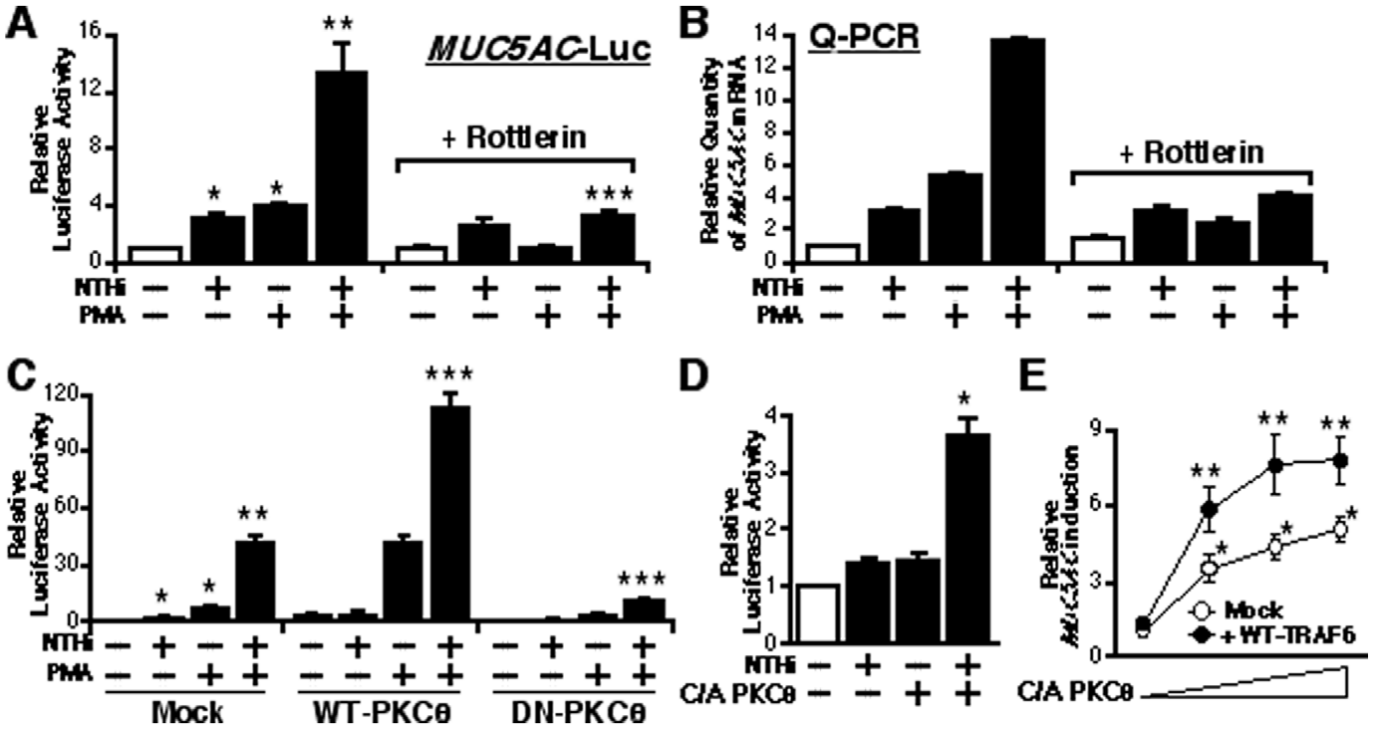
TLR-TRAF6-dependent synergistic MUC5AC induction by NTHi and PMA is mediated by PKCθ. (**A**) Rottlerin, a specific PKC™/θ inhibitor, blocked the synergistic induction of MUC5AC transcription by NTHi and PMA in human epithelial cells, as assessed by MUC5AC-dependent promoter Luciferase assay. Cells were pre-treated with 20 µM of Rottlerin or vehicle control, and treated with NTHi with or without PMA. Relative luciferase activity of MUC5AC was measured from the cell lysate. Values are the means ± S.D. (n = 3). *p<0.05 vs. control; **p<0.05 vs. NTHi alone; ***p<0.05 vs. NTHi with PMA in vehicle treated cells. (**B**) The synergistic induction of MUC5AC expression was also attenuated by Rottlerin at the mRNA level, as assessed by performing Q-PCR. Cells were pre-treated with 20 µM of Rottlerin or vehicle control, and treated with NTHi with or without PMA. mRNA expression level of MUC5AC was measured by Q-PCR. (**C**) Co-expressing WT-PKCθ enhanced, whereas DN-PKCθ inhibited, the synergistic induction of MUC5AC transcription by NTHi and PMA. Cells were transfected with 0.3 µg of WT- PKCθ, 0.6 µg of DN PKCθ, or control vector, and treated with NTHi with or without PMA. Relative luciferase activity of MUC5AC was measured from the cell lysate. Values are the means ± S.D. (n = 3). *p<0.05 vs. control; **p<0.05 vs. NTHi alone; ***p<0.05 vs. NTHi with PMA in control vector transfected cells. (**D**) C/A-PKC-induced MUC5AC expression was synergistically enhanced by NTHi in human epithelial cells. Cells were transfeced with 0.3 µg of C/A PKCθ or control vecttor, and treated with NTHi. Relative luciferase activity of MUC5AC was measured from the cell lysate. Values are the means ± S.D. (n = 3). *p<0.05 vs. NTHi alone. (**E**) C/A-PKCθ synergized with WT-TRAF6 to induce MUC5AC expression in a dose-dependent manner. Cells were transfected with 0.1, 0.3, or 0.6 µg of C/A PKCθ with or without 0.3 µg of WT-TRAF6. mRNA expression level of MUC5AC was measured by Q-PCR. Values are the means ± S.D. (n = 3). *p<0.05 vs. control; **p<0.05 vs. C/A PKCθ transfected cells. The data shown are representative of three independent experiments. −, absence of; +, presence of; NTHi, nontypeable *Haemophilus influenzae*; WT, wild-type; DN, dominant negative; C/A, constitutively active form.

Although we have demonstrated that NTHi-induced TLR2-TRAF6-dependent MKK3/6-p38 MAPK pathway is synergistically enhanced by PMA to up-regulate MUC5AC expression, it is still unclear how PMA transduces signal to TLR-dependent pathway. It has been known that PMA modulates diverse cellular responses such as gene transcription, cellular growth and differentiation, programmed cell death, the immune response, and receptor desensitization through PKC signaling pathway [79], [80]. Among various PKC isoforms, PKCθ has recently been shown to play an important role in PMA-induced MUC5AC up-regulation [35], [63]. To determine whether PKCθ is involved in the synergistic enhancement of MUC5AC induction by NTHi and PMA, we first assessed the effect of perturbing PKC signaling on MUC5AC expression. As shown in **Fig. 6A and B**, Rottlerin, a specific PKC™/θ inhibitor, abolished PMA-induced synergistic enhancement of MUC5AC expression by NTHi at both the transcriptional and mRNA levels, as assessed by performing Luciferase assay and Q-PCR, respectively. To define the involvement of PKCθ in synergistic MUC5AC induction, we next evaluated the effect of co-expressing either wild-type (WT) or DN mutant form of PKCθ on MUC5AC-dependent promoter activity. As shown in **Fig. 6C**, co-expression of WT-PKCθ further enhanced, whereas co-expression of DN-PKCθ attenuated NTHi- and PMA-induced MUC5AC-Luc promoter activity. In addition, NTHi-induced MUC5AC transcription was synergistically enhanced by co-expression of constitutively-active (C/A) form of PKCθ (C/A-PKCθ), instead of PMA treatment (**Fig. 6D**). Moreover, C/A-PKCθ itself also synergistically enhanced WT-TRAF6-induced MUC5AC transcription in a dose-dependent manner (**Fig. 6E**), providing supportive evidence that PMA-induced PKCθ signaling modulates TLR-dependent MUC5AC induction via TRAF6. Collectively, we concluded from these data that PMA synergises with NTHi to induce TLR-TRAF6-dependent MUC5AC induction via PKCθ.

### CARMA1 acts downstream of PKCθ in mediating PMA-induced synergistic enhancement of TLR-TRAF6-dependent MUC5AC expression

**Figure 7:**
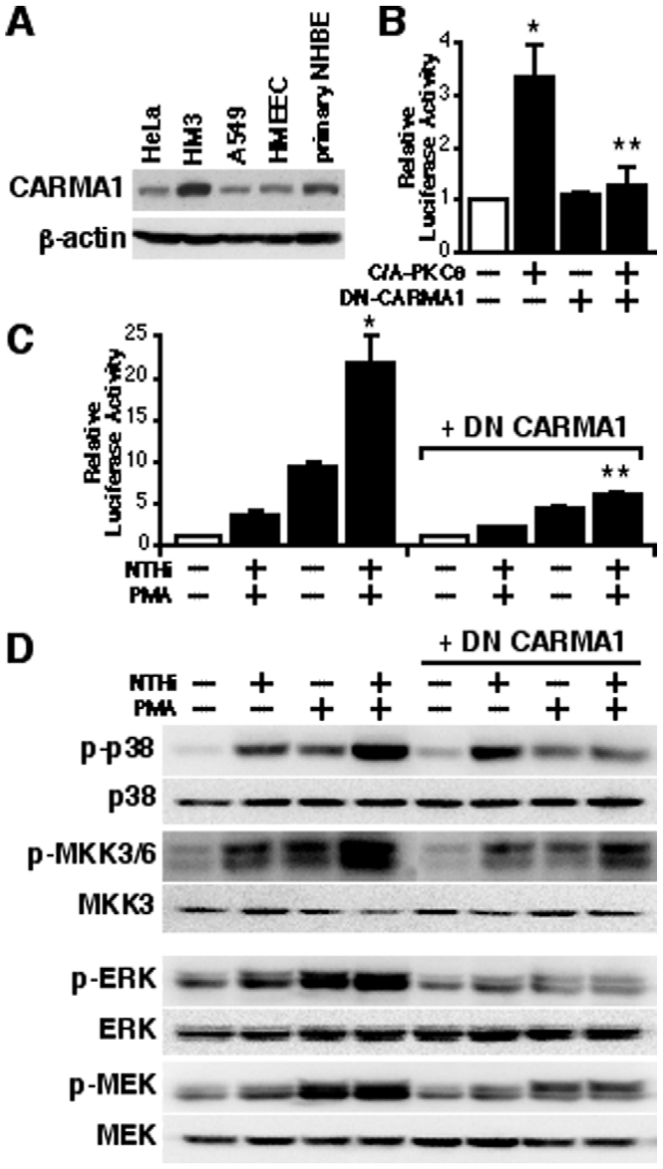
CARMA1 acts downstream of PKCθ in mediating PMA-induced synergistic enhancement of TLR-TRAF6-dependent MUC5AC expression. (**A**) CARMA1 was expressed in a variety of epithelial cells, such as in human cervix HeLa, colon HM3, airway A549, middle ear HMEEC-1 and primary bronchial epithelial NHBE cells, as assessed by WB using antibody against CARMA1. (**B**) Overexpression of a DN mutant form of CARMA1 attenuated C/A-PKCθ-induced MUC5AC expression. Cells were transfeced with 0.3 µg of C/A PKCθ with or without 0.8 µg of DN-CARMA1. Relative luciferase activity of MUC5AC was measured from the cell lysate. Values are the means ± S.D. (n = 3). *p<0.05 vs. control; **p<0.05 vs. C/A PKCθ transfected cells. (**C**) The synergistic induction of MUC5AC transcription by NTHi and PMA was potently inhibited by overexpression of DN-CARMA1. Cells were transfeced with 0.8 µg of DN-CARMA1 or control vector, and treated with NTHi with or without PMA. Relative luciferase activity of MUC5AC was measured from the cell lysate. Values are the means ± S.D. (n = 3). *p<0.05 vs. NTHi alone; **p<0.05 vs. control vector transfected cells. (**D**) Overexpression of a DN mutant form of CARMA1 greatly inhibited not only synergistic phosphorylation of p38 MAPK and MKK3/6 induced by NTHi and PMA, but also ERK and MEK phosphorylation. Cells were transfeced with 0.8 µg of DN-CARMA1 or control vector, and treated with NTHi with or without PMA. Cell lysate was blotted with antibodies indicated in the figure. The data shown are representative of three independent experiments. −, absence of; +, presence of; NTHi, nontypeable *Haemophilus influenzae*; DN, dominant negative; C/A, constitutively active form.

Having identified the requirement of PKCθ for synergistic MUC5AC induction, it is still unknown which intermediate signaling molecule links PKCθ signaling to TLR-dependent pathway. Recently, interesting studies have demonstrated that CARMA1, also known as CARD11, acts downstream of PKCθ to regulate the adaptive immune response in T cells [46], [47], [48], [51], [54], [78], [81]. To determine the possible involvement of CARMA1 in PMA-induced synergistic enhancement of MUC5AC induction, we first determined whether CARMA1 is expressed in a variety of human epithelial cells by Western blotting using antibody against CARMA1. As shown in **Fig. 7A**, CARMA1 is expressed in human cervix epithelial HeLa, colon epithelial HM3, airway epithelial A549, and middle ear epithelial HMEEC-1 cell lines as well as primary bronchial epithelial NHBE cells. As expected, C/A-PKCθ-induced MUC5AC transcription was abolished by co-expressing a DN form of CARMA1, indicating that CARMA1 indeed acts downstream of PKCθ (**Fig. 7B**). We then assessed the effect of co-expressing DN-CARMA1 on the synergistic enhancement of MUC5AC expression. As shown in **Fig. 7C**, co-expressing DN-CARMA1 markedly inhibited the synergistic induction of MUC5AC expression by NTHi and PMA. Concomitantly, overexpression of DN-CARMA1 greatly inhibited not only the synergistic phosphorylation of p38 MAPK and MKK3/6 induced by NTHi and PMA, but also the phosphorylation of ERK and MEK1 (**Fig. 7D**). Thus, these data indicate that CARMA1 acts downstream of PKCθ in mediating PMA-induced synergistic enhancement of TLR-TRAF6-dependent MUC5AC expression.

### CARMA1 mediates TLR-dependent synergistic MUC5AC induction by NTHi and PMA via TRAF6

**Figure 8:**
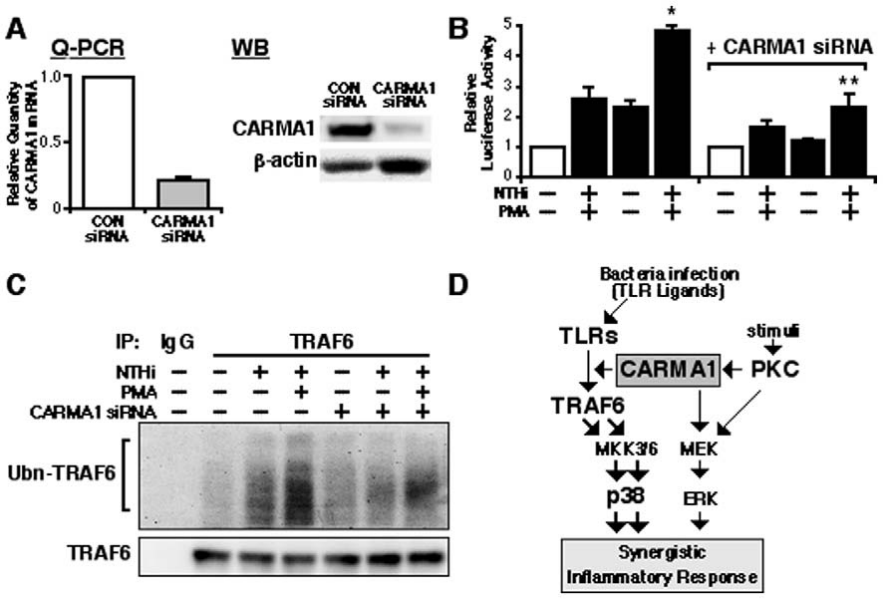
CARMA1 mediates TLR-dependent synergistic MUC5AC induction by NTHi and PMA via cross-talk with TRAF6. (**A**) CARMA1 knockdown by CARMA1-siRNA efficiently reduced the endogenous CARMA1 expression at both the mRNA and protein level, as assessed by Q-PCR and WB, respectively. (**B**) CARMA1-siRNA inhibited the synergistic induction of MUC5AC transcription by NTHi and PMA. Values are the means ± S.D. (n = 3). *p<0.05 vs. NTHi alone; **p<0.05 vs. control siRNA transfeced cells. (**C**) Synergistic enhancement of NTHi-induced TRAF6 polyubiquitination by PMA was attenuated by CARMA1 knockdown. (**D**) Schematic representation depicting how CARMA1 mediates the synergistic enhancement of *MUC5AC* expression in human epithelial cells. The data shown are representative of three independent experiments. CON, control; −, absence of; +, presence of; NTHi, nontypeable *Haemophilus influenzae*; Ubn, ubiquitin; TLRs, toll-like receptors; TRAF6, TNF receptor associated factor 6; PKC, protein kinase C.

To further define the mechanism by which CARMA1 enhances TLR-TRAF6-dependent signaling, we determined the involvement of CARMA1 by using siRNA approach. As expected, a CARMA1-specific small interfering RNA (CARMA1-siRNA) efficiently reduced endogenous CARMA1 expression at both the mRNA and protein levels, as assessed by performing Q-PCR and Western blotting (**Fig. 8A**). Consistent with the results shown in **Fig. 7**, CARMA1 knockdown by siRNA greatly inhibited the synergistic induction of MUC5AC transcription by NTHi and PMA (**Fig. 8B** **)**, providing supporting evidence for the critical involvement of CARMA1 in mediating the synergistic MUC5AC induction. We next sought to explore how CARMA1 mediates TLR-TRAF6-dependent pathway. Recent study has shown that CARMA1 mediates PKCθ-induced polyubiquitination of TRAF6 to activate NF-κB in T cells [82]. Moreover, our data (Fig. 5) showed that NTHi and PMA synergistically induced polyubiquitination of TRAF6. Thus we evaluated the effect of CARMA1-siRNA on TRAF6 polyubiquitination induced by NTHi and PMA. The synergistic enhancement of NTHi-induced TRAF6 polyubiquitination by PMA was markedly attenuated by CARMA1 knockdown (**Fig. 8C**). Taken together, our data demonstrated that CARMA1 mediates TLR-dependent synergistic MUC5AC induction by NTHi and PMA via TRAF6. The functional involvement of TRAF6 ubiquitination in mediating the synergistic induction of MUC5AC needs to be further investigated in future studies.

## Discussion

In the present study, we showed that phorbol ester (PMA) synergizes with bacterium NTHi to induce up-regulation of MUC5AC expression, which is known to play an important role in mucosal defense against invading bacterial pathogens. PMA-induced synergistic enhancement of MUC5AC expression is mediated by TLR2-dependent activation of TRAF6-MKK3/6-p38 MAPK signaling pathway. PKCθ modulates TLR-TRAF6-dependent signaling to synergistically enhance MUC5AC induction by NTHi and PMA. Moreover, CARMA1, an important signaling mediator upon TCR activation in T cells, acts downstream of PKCθ to mediate TLR-dependent synergistic MUC5AC induction via TRAF6 (**Fig. 8D**).

Of particular interest in this study is that PKCθ modulates bacteria-induced TLR2-TRAF6-dependent signaling pathway to synergistically enhance MUC5AC mucin up-regulation, a primary innate defense response for mammalian airways. Although extensive efforts have been made towards understanding the critical role for PKC in inflammatory responses, still unknown is the role of PKC in mediating host mucosal innate immune responses in chronic inflammatory and infectious diseases, such as COPD and OM. In the present study, our data clearly indicate that phorbol ester PMA, a potent PKC activator and an analogues of diacylglycerol (DAG), synergizes with bacterium NTHi to induce MUC5AC expression via cross-talk with TRAF6. Interestingly, the synergistic effect of PMA appers to be specific only for TLR-dependent signaling pathway (**Fig. 4**). Furthermore, similarly to MUC5AC up-regulation, PMA also synergistically enhance NTHi-induced expression of pro-inflammatory cytokines, such as TNF-α, IL-1β and IL-8 (data not shown). These results, although rather unexpected, may provide novel implication into the role of PKC in regulating TRAF6-dependent mucosal innate immune responses. There is now accumulating evidence to suggest that activation of PKC isotypes is induced by multiple stimuli, such as LPS, neutrophil elastase, nicotine and cigarette smoke [83], [84], [85], [86]. Those stimuli have also been widely known as the major cause of chronic inflammatory and infectious diseases, such as COPD and OM [87], [88], [89]. Therefore, under diseased conditions such as COPD and OM, it is anticipated that activation of PKC may contribute to the detrimental and overactive host responses, such as mucin overproduction, especially in infectious diseases. Thus, our data reveal a novel role of PKCθ in regulating the host mucosal innate immune response in the pathogenesis of chronic inflammatory and infectious diseases.

Another important finding in the present study is the direct evidence showing that CARMA1 acts downstream of PKCθ to mediate TLR-dependent synergistic MUC5AC induction via TRAF6 in human epithelial cells. CARMA1 contains a CARD and a MAGUK domain, and plays an essential role in the adaptive immune response [49], [50], [90], [91], [92]. CARMA1 also functions as a molecular scaffold in the assembly of multi-protein complexes and has recently been implicated in signaling from PKC to NF-κB activation in T cells [46], [47], [48], [51], [52], [55]. It has been previously reported that CARMA1 is predominantly expressed in spleen, thymus, and peripheral blood leukocyte, associates with Bcl10-MALT1 complex and recruits these proteins into the lipid rafts by an unknown mechanism [50], [87], [93], [94]. The recruitment of the Bcl10-MALT1 complex has been shown to activate IKK through an ubiquitin-dependent pathway leading to activation of NF-κB [47], [52], [55], [95], [96]. Although several studies have demonstrated the critical role for CARMA1 in the adaptive immune response in T cells, little is known about the function of CARMA1 in epithelial cells. Moreover, the involvement of CARMA1 in bacteria-induced host defense has yet to be addressed. As evidenced by the data shown in **Fig. 7** and **8**, CARMA1 is expressed in a variety of human epithelial cell lines as well as primary bronchial epithelial cells, and mediates bacteria-induced TLR-dependent synergistic MUC5AC up-regulation (**Fig. 7**). Furthermore, CARMA1 acts downstream of PKCθ to modulate TLR-dependent signaling via TRAF6 (**Fig. 8**). Given that inhibition of CARMA1 blocked not only the synergistic activation of MKK3/6-p38 MAPK pathway but also activation of MEK-ERK by PMA, it is likely that CARMA1 plays a central role in this synergistic induction of MUC5AC. Thus, our studies provide direct evidence for the critical involvement of CARMA1 in bacteria-induced host defense especially in human epithelial cells, thus bringing new insights into the novel molecular function of CARMA1 in regulating host mucosal innate immune response in respiratory epithelial surface, the first line of host defense.

## Materials and Methods

### Reagents

Recombinant human TNF-α and IL-6 were purchased from R&D Systems. PGN, Zymosan, Poly(I:C) and LPS were purchased from InvivoGen (San Diego, CA). PMA, SB203580 and Rottlerin were purchased from Calbiochem (LaJolla, CA).

### Cell Culture

HM3 (human colon epithelial), HeLa (human cervix epithelial), A549 (Human lung epithelial), and HMEEC-1 (human middle ear epithelial) cells were maintained and used as described previously [5], [6], [74], [76], [77], [97], [98]. HM3 and HMEEC-1 cells are from Dr. Y.S. Kim and Dr. David Lim, respectively, as described previously [40], [71], [99], [100]. HeLa and A549 cells are from ATCC. All media contained 10% fetal bovine serum (Invitrogen), penicillin (100 U/ml) and streptomycin (0.1 mg/ml). All cells were cultured in a humidified atmosphere of 5% CO_2_ at 37°C. Primary human bronchial epithelial cells (NHBE) were purchased from Clonetics (San Diego, CA) and maintained in Clonetics' recommended bronchial epithelial growth media (BEGM) following the manufacturer's instructions [5], [6], [76], [98]. Stable cell lines, HEK293-pcDNA and HEK293-TLR2 were kindly provided by D. T. Golenbock as described previously [97], [101]. Wild-type (WT) and TLR2^−/−^ MEFs were obtained from E13 embryos of WT and TLR2^−/−^ mice, respectively and maintained in DMEM supplemented with 10% fetal bovine serum (Invitrogen, Carlsbad, CA, USA) as described previously [74]. TLR2^−/−^ mice have been reported previously and provided by Dr. Shizuo Akira [9], [102]. Isolation and culture of MEF cells from TLR2^−/−^ mice has been approved the by the Institutional Animal Care and Use Committee at University of Rochester.

### Bacterial Strain and Culture

NTHi strain 12, a clinical isolate, was used in this study [8], [9], [40], [41], [74], [76]. Bacteria were grown on chocolate agar at 37°C in an atmosphere of 5% CO_2_. For making NTHi crude extract, NTHi were harvested from a plate of chocolate agar after overnight incubation and incubated in 30 ml of brain heart infusion (BHI) broth supplemented with NAD (3.5 g/ml). After overnight incubation, NTHi were centrifuged at 10,000 g for 10 min, and the supernatant was discarded. The resulting pellet of NTHi was suspended in 10 ml of phosphate-buffered saline and sonicated. Subsequently, the lysate was collected and stored at 70°C. NTHi lysates (5 µg/ml) were used in all the experiments. *S.p.* strain D39 was used and crude extracts were used as described [70], [71], [74], [77].

### Plasmids, Transfection and Luciferase assay

The plasmids fp38α (AF), fp38β_2_ (AF), WT-TRAF6, Dominant-negative (DN)-TRAF6 (TRAF6:299–522), DN-TLR2, WT-PKCθ, DN-PKCθ (K409R), constitutively-active (C/A)-PKCθ (A148E), and MUC5AC-luciferase reporter were described previously [5], [6], [8], [9], [40], [41], [68], [70], [71], [72], [76]. DN-CARMA1 was kindly provided by Dr. Xin Lin [44], [45]. Empty vector was used as a control. All transient transfections were carried out in triplicate using a TransIT-LT1 reagent (Mirus, Madison, WI) following the manufacturer's instructions. At 40 h after starting the transfection, cells were pretreated with or without chemical inhibitors including SB203580 or Rottlerin for 1 h. NTHi and PMA were then added to the cells for 5 h before cell lysis for Luciferase assay.

### Small-interfering RNA (siRNA)

CARMA1-siRNA and siCONTROL (non-targeting siRNA Pool) were purchased from Dharmacon. Cells were transfected with a final concentration of 100 nM siRNA using Lipofectamine 2000 (Invitrogen Life Technologies). Forty hours after the start of transfection, cells were treated with NTHi and PMA as indicated before lysed for Luciferase assay and Immunoprecipitation.

### Real-time Quantitative PCR (Q-PCR) Analysis

Total RNA was isolated using TRIzol reagent (Invitrogen) following the manufacturer's instructions. For the reverse transcription reaction, TaqMan reverse transcription reagents (Applied Biosystems) were used. Briefly, the reverse transcription reaction was performed for 60 min at 37°C, followed by 60 min at 42°C by using oligo(dT) and random hexamers. PCR amplification was performed by using TaqMan Universal Master Mix. In brief, reactions were performed in duplicate containing 2×Universal Master Mix, 1 µl of template cDNA, 100 nM primers, and 100 nM probe in a final volume of 12.5 µl, and they were analyzed in a 96-well optical-reaction plate (Applied Biosystems). Probes for TaqMan include a fluorescent reporter dye, 6-carboxyfluorescein (FAM), on the 5′ end and labeled with a fluorescent quencher dye, 6-carboxytetramethylrhodamine (TAMRA), on the 3′ end to allow direct detection of the PCR product. Reactions were amplified and quantified by using as ABI 7700 sequence detector and the manufacturer's corresponding software (Applied Biosystems). Relative quantity of mRNAs were obtained by using the comparative Ct Method (for details, see User Bulletin 2 for the ABI PRISM 7500 sequence-detection system) and was normalized by using TaqMan Pre-Developed Assay Reagent human cyclophilin as an endogenous control (Applied Biosystems). The primers and probe for human MUC5AC were as follows: 5′-GTT CTA TGA GGG CTG CGT CTT T-3′ (forward primer) and 5′-GGC TGG AGC ACA CCA CAT C-3′ (reverse primer); TaqMan probe, 5′-FAM-ACC GGT GCC ACA TGA CGG ACC T-TAMRA-3′.

### Western Blot (WB) Analysis

Western blots were performed as described [97]. Briefly, western blots were performed using whole cell extracts, separated on 8–10% SDS-PAGE gels and transferred to polyvinylidine difluoride membranes (Pall Life Sciences, Pensacola, FL). The membrane was blocked with a solution of TBS containing 0.1% Tween 20 (TBS-T) and 5% nonfat milk. After three washes in TBS-T, the membrane was incubated in a 1∶1000 dilution of a primary antibody. After another three washes in TBS-T, the membrane was incubated with 1∶2000 dilution of the corresponding secondary antibody. The membrane was reacted with chemiluminescence reagent ECL (Amersham Biosciences) to visualize to blots. Antibodies against phospho-p38 (Thr180/Tyr182), p38, phospho-MKK3/6 (Ser-189/207), MKK3, phospho-ERK1/2 (Thr202/Tyr204), ERK1/2, phospho-MEK1/2 (Ser217/221), MEK1/2 and CARMA1 were purchased from Cell Signaling Technology (Beverly, MA), Antibodies against TRAF6 and Ubiquitin were from Santa Cruz Biotechnology, and anti-β-actin was from Sigma.

### Immunoprecipitation (IP)

For immunoprecipitation, 800 µl of lysates were incubated for 1 hour at 4°C with control mouse IgG and protein A/G-agarose (Santa Cruz Biotechnology). After centrifugation, anti-TRAF6 antibody (Santa Cruz Biotechnology) was incubated with supernatant for 1 h at 4°C, followed by incubation overnight with protein A/G-agarose. Immunoprecipitates were washed twice with RIPA buffer, resuspended in 2× SDS loading buffer, and separated on 8% SDS-PAGE, followed by Western blot analysis.
